# Herd-level risk factors associated with *Leptospira *Hardjo seroprevalence in Beef/Suckler herds in the Republic of Ireland

**DOI:** 10.1186/2046-0481-65-6

**Published:** 2012-03-26

**Authors:** Eoin Gerard Ryan, Nola Leonard, Luke O'Grady, Michael L Doherty, Simon J More

**Affiliations:** 1 School of Veterinary Medicine, University College Dublin, Belfield, Dublin 4, Republic of Ireland

**Keywords:** Leptospirosis, Hardjo, Suckler, Ireland, Risk factors, Questionnaire, Herd size, Region

## Abstract

**Background:**

The aim of the present study was to investigate risk factors for herd seropositivity to *Leptospira *Hardjo in Irish suckler herds. Herds were considered eligible for the study if they were unvaccinated and contained ≥ 9 breeding animals of beef breed which were ≥ 12 months of age. The country was divided into six regions using county boundaries. Herd and individual animal prevalence data were available from the results of a concurrent seroprevalence study. Herds were classified as either "Free from Infection" or "Infected" based on a minimum expected 40% within-herd prevalence.

Questionnaires were posted to 320 farmers chosen randomly from 6 regions, encompassing 25 counties, of the Republic of Ireland. The questionnaire was designed to obtain information about vaccination; reproductive disease; breeding herd details; the presence of recognized risk factors from previous studies; and husbandry on each farm. Data collected from 128 eligible herds were subjected to statistical analysis.

**Results:**

Following the use of Pearson's Chi-Square Test, those variables associated with a herd being "infected" with a significance level of P < 0.2 were considered as candidates for multivariable logistic regression modelling. Breeding herd size was found to be a statistically significant risk factor after multivariable logistic regression. The odds of a herd being positive for leptospiral infection were 5.47 times higher (P = 0.032) in herds with 14 to 23 breeding animals compared with herds with ≤ 13 breeding animals, adjusting for Region, and 7.08 times higher (P = 0.033) in herds with 32.6 to 142 breeding animals.

**Conclusions:**

Breeding herd size was identified as a significant risk factor for leptospiral infection in Irish suckler herds, which was similar to findings of previous studies of leptospirosis in dairy herds.

## Background

Leptospirosis, due to *Leptospira *Hardjo, is a disease of cattle worldwide [1-7]. In Ireland there are two species of leptospires that are associated with disease: *Leptospira interrogans *serovar Hardjo and *Leptospira borgpetersenii *serovar Hardjo. Collectively, both species can be referred to as *Leptospira *Hardjo. *L*. Hardjo mainly causes reproductive disease, i.e. abortion, mummification, stillbirth, premature and term birth of weak calves [8-11], as well as causing milk drop syndrome in dairy herds [12,13]. Leptospirosis is recognised as a significant zoonotic disease of farmers, farm workers and workers involved in the agricultural industry [14-19].

Herd-level risk factors for leptospirosis due to *L*. Hardjo in dairy herds include: larger herd size; co-grazing with infected cattle or sheep; access of cattle to contaminated water courses; use of a stock bull; inadequate husbandry practices and purchase of replacement breeding animals [4,11,20-23]. In an Irish study of unvaccinated dairy herds, [24], both the probability of a herd being seropositive and the antibody level in the herd milk sample were affected by the province and the herd size category. Larger herds were significantly more likely to have positive reactions and higher mean concentrations of bulk milk antibody. The risk factors for leptospirosis in suckler herds are less well established than for dairy herds, and no previous studies have reported prevalence or risk factors for leptospirosis in Irish suckler herds.

The aim of the present study was to investigate risk factors for herd seropositivity to *Leptospira *Hardjo in Irish suckler herds.

## Materials and methods

### Study design

This cross-sectional epidemiological study was carried out in the School of Agriculture, Food Science & Veterinary Medicine, University College Dublin, together with a seroprevalence study of *Leptospira *Hardjo in Irish suckler/beef herds [25]. The herds included in this study were a random subset of herds chosen as part of a paratuberculosis research project in Ireland [26] and, therefore, no additional ethical approval was required in order to use the selected serum samples. This population of herds was a subset of the national herd as chosen randomly from the herds tested for brucellosis in 2004 and 2005 under the National Brucellosis Eradication Scheme. They consisted of 1,000 herds (mixed suckler and dairy) randomly chosen from an eligible total of 96,163 herds where at least one calf had been registered on the Cattle Movement Monitoring System (CMMS) as born in the herd in 2003 [27].

**Figure 1:**
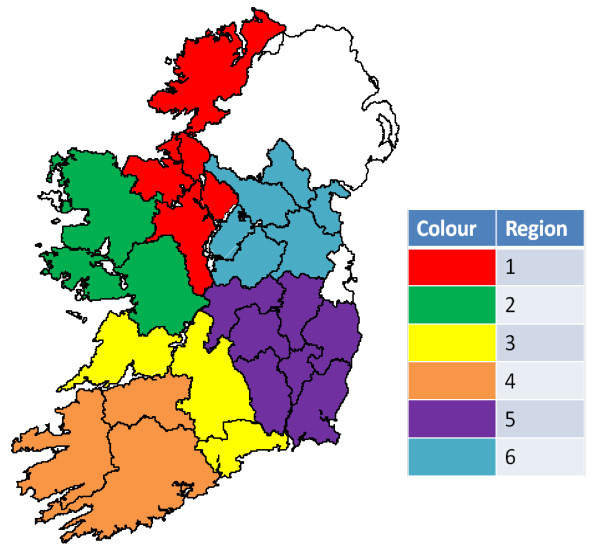
**Regions in the Republic of Ireland used in the *Leptospira *Hardjo seroprevalence **[25]**and risk factor studies**.

The *Linnodee Leptospira ELISA Kit™ *(Linnodee Animal Care, Ballyclare, Northern Ireland) [28-30] was used to test all serum samples [25]. This ELISA detects an IgG antibody response to a lipopolysaccharide outer envelope epitope common to both *Leptospira borgpetersenii *serovar Hardjo and *Leptospira interrogans *serovar Hardjo [30]. Participating herds in the study had (i) to be unvaccinated, (ii) to contain ≥ 9 eligible breeding animals (bulls and breeding females ≥ 12 months of age) and (iii) to have breeding animals of beef breeds only. The country was divided into six regions using county boundaries (Region 1--North West; Region 2--West Connaught; Region 3--North Munster; Region 4--South West Munster; Region 5--South East Leinster; Region 6--North Leinster/South Ulster) (Figure 1) [25]. Each region had approximately equal numbers of suckler cows based on data from the Central Statistics Office Census of Agriculture, 2000 [31]. Herds and animals were chosen randomly from each region for inclusion in the study.

### Assigning herd infection status

Herd-level seroprevalence was determined after first defining each study herd as "infected" or not, based on the serological results obtained. A programme, *FreeCalc 2.0 *[32-34], was used during herd classification, calculating the probability of freedom from infection in each study herd, given the test results, the likely minimum herd prevalence assuming infection, the limitations of the serological test (in particular, imperfect specificity leading to false positive results) and after accounting for finite herd size. The methodology is a probabilistic approach to this problem, with the application of a hypergeometric exact probability formula and a result expressed in terms of probability of freedom. The following data were used during these calculations: test (ELISA) sensitivity and specificity, estimated minimum expected (within-herd) infection prevalence, and population (herd) size. Herd-level sensitivity (HSENS) and herd-level specificity (HSPEC) were chosen to be 95% respectively. Based on knowledge of the biology of the disease [35], on published within-herd prevalence rates in endemic herds (41.8% [21]; 62% [36]), and using a trial and error approach, it was found that a within-herd prevalence of 40% allowed the rejection of the null hypothesis (null hypothesis = herds are infected) when sampling a maximum of 20 animals per herd, using the ELISA with test sensitivity of 100% and test specificity of 86.67%. For herds of < 20 eligible breeding animals, all animals were sampled. Ultimately, all herds were classified as either "Free from Infection" or "Infected" at the 95% confidence interval at a within-herd prevalence of 40%.

The apparent within-herd prevalence of each herd was calculated by expressing the number of ELISA-positive animals as a percentage of the total number of animals sampled in the herd. Estimated true within-herd prevalence, at the 95% confidence interval, was then calculated by using the epidemiological computer software tool, *TruePrev *[37], which takes into account the sensitivity and specificity of the test used and the number of animals tested.

### The questionnaire

Once the participating herds had been chosen, a questionnaire was created in order to investigate the potential risk factors for herd seropositivity to *Leptospira *Hardjo. The risk factors chosen were broadly based on the results of similar studies in relation to dairy herds [22]. In March 2005, a small pilot study was completed on 10 dairy herds in Co. Wicklow in order to refine and focus the questions included in the questionnaire. The aim was to have the majority of questions as unambiguous as possible with closed (either "Yes" or "No") answers.

**Figure 2:**
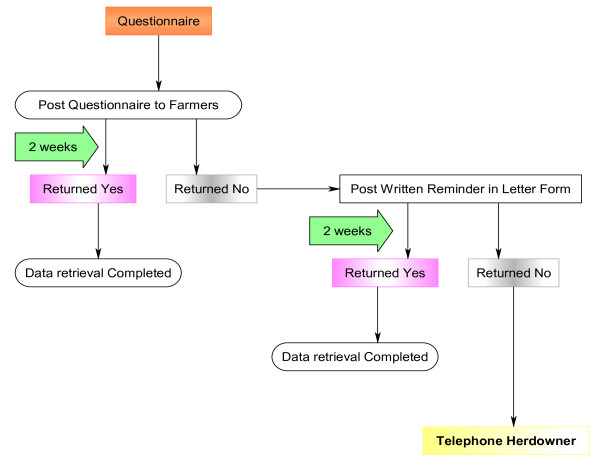
**Flow diagram illustrating *Leptospira *Hardjo questionnaire timeline**.

Figure 2, is a diagrammatic representation of the timeline involved in posting out the questionnaire to the chosen herd-owners in May, July and August 2005 and the subsequent steps towards data retrieval.

All herds that did not respond to the questionnaire (n = 163) were contacted by telephone to establish the leptospirosis vaccination status of each herd.

Due to the lack of data in relation to particular questions, not all of the listed questions were included in the final dataset for statistical analysis.

### Data collection/management

The results from each question in the questionnaire were compiled in an Excel spreadsheet. To aid in the statistical analysis of the available data, the following variables were categorized: Region (1-6); Breeding Herd Size (quartiles); Grazing Acres (1 acre = 0.405 hectares); Percentage of Grazing Area Wet. As insufficient data were available in association with the following questions, the data were either included in an altered form or were excluded from the statistical analysis: "Other Disease History" was excluded; "Cow Abortions 2003" and "Heifer Abortions 2003" were amalgamated to one variable of "Abortions 2003" and a similar process was used to create "Abortions 2004" and "Abortions 2005"; the variable "Bull Yes/No" was created by amalgamating "Stock Bull" and "Hired/Borrowed Bull" figures.

Data were managed using Microsoft Excel (Microsoft Office 2007, Microsoft Corporation, Redmond, Washington, USA) and transferred using Stat Transfer 8.2 to Stata SE V10.0 (StataCorp, Texas, USA).

### Data analysis

#### Univariate study methodology

The Pearson's Chi-square test was used to assess the unadjusted relationship between herd leptospirosis status ("Free from Infection" or "Infected") and a number of associated variables (n = 62). Similar analysis was conducted to assess the unadjusted relationship between Region and these variables arising from the questionnaire. Univariate statistical analysis, to a level of P < 0.05, was carried out on the 14 significant variables (P < 0.2) from the Chi-squared analysis.

#### Multivariable modelling methodology

Those variables associated with a herd being positive for leptospiral infection with a significance level of P < 0.2 following Chi-squared analysis were considered as candidates for multivariable modelling. Spearman correlation analyses were conducted to check for potential collinearity (r > |0.6|) between the independent variables. For pairs of variables that showed collinearity, one of the predictor variables was selected for inclusion in the final analyses, and the other ignored [38]. A multivariable logistic regression model was fitted to assess the relationship between herd leptospirosis status and a number of variables. Models were built using backward elimination. The fit of the model was examined using the Hosmer-Lemeshow goodness of fit *X*^2 ^(p = 0.6886).

## Results

### The study herds

In total, 157 completed/partially completed questionnaires were returned. Twenty-one herds were vaccinating against leptospirosis and a further 7 herds were excluded because they had < 9 eligible breeding animals in the herd. One herd was removed from the dataset due to lack of information supplied in the returned questionnaire. Ultimately, 128 herds were included in the final risk factor survey (Table 1). The questionnaire response rate was 157/320 herds or 49.06%.

**Table 1:** Summary of herds included in risk factor analysis for leptospiral infection

	Farmers receiving questionnaire	Questionnaire returned	Farmers vaccinating	Herds ≤ 8 Breeding Animals	Insufficient data	Number of herds in final dataset
Region 1	65	35	3	3	0	**29**
Region 2	63	35	6	2	0	**27**
Region 3	51	18	1	0	1	**16**
Region 4	51	18	3	1	0	**14**
Region 5	50	27	4	0	0	**23**
Region 6	40	24	4	1	0	**19**
Totals	320	157	21	7	1	**128**

### Descriptive results

The seroprevalence of *Leptospira *Hardjo in the 128 herds included in the risk factor study is shown in Table 2. The prevalence distribution of the risk factor (RF) herds is representative of the overall herd prevalence of the 288 herds involved in the original seroprevalence study (SP). Overall herd prevalence is very similar for the herds that did (RF) (83.59%) and did not (NR) (81.25%) respond to the questionnaire, indicating that there is no evidence of significant bias between the two subsets of herds. The median breeding herd sizes (BHS) for each Region are also displayed in Table 2. It can be seen that median BHS for the RF herds is highest in regions 5 and 6, and smallest in region 2. This is broadly in keeping with the herd prevalence in these regions. It can be seen from Table 2 that BHS overall does not differ significantly between RF herds and NR herds.

**Table 2:** Herd Prevalence of Leptospira Hardjo (%) and Median Breeding Herd Size (BHS) by Region: Herds included in a Risk Factor (RF) Study (n = 128); Seroprevalence (SP) Study (n = 288) and Herds Failing to Return Questionnaire (NR) (n = 160)

Region	(RF) Herds Free from Infection	(RF) Herds Infected	(RF) Total Herds	(RF) Herd Prevalence %	(SP) Herd Prevalence%	(NR) Herd Prevalence %	(RF) Median BHS	(NR) Median BHS
Region 1	6	23	29	**79.31**	**82.76 (n = 58)**	**86.21 (n = 29)**	20.00	21.00
Region 2	8	19	27	**70.37**	**75.93 (n = 54)**	**81.48 (n = 27)**	19.00	20.00
Region 3	4	12	16	**75.00**	**80.00 (n = 50)**	**82.35 (n = 34)**	22.50	28.50
Region 4	1	13	14	**92.86**	**85.11 (n = 47)**	**81.82 (n = 33)**	22.00	21.00
Region 5	0	23	23	**100.00**	**93.33 (n = 45)**	**86.36 (n = 22)**	28.50	26.50
Region 6	2	17	19	**89.47**	**76.47 (n = 34)**	**60.00 (n = 15)**	28.00	18.00
**Totals**	**21**	**107**	**128**	**83.59**	**82.29 (n = 288)**	**81.25 (n = 160)**	**23.33**	**22.00**

In a comparison of the 21 vaccinating herds and the 128 risk factor herds, many variables showed markedly contrasting findings for the two groups. While there were only 3% of herds in the unvaccinated group with a history of leptospirosis, there were 52.38% in the vaccinating group. The vaccinated herds had a much higher incidence of abortions, stillbirths, weak calves and apparent infertility (difficulty getting cows in-calf). BHS was also markedly different between the two groups with the vaccinating herds having a much larger mean BHS. Vaccinated herds were more likely to buy in replacement breeding animals and to have part of their grazing area flooded each year.

### Data analysis

Following Chi-squared testing, there were 14 significant variables (P < 0.2) remaining (Table 3).

**Table 3:** The 14 Variables with P < 0.2 following analysis using the Chi-Square Test to assess the relationship between Herd Leptospirosis Status and 62 Variables in 128 herds

		Leptospirosis Status		
**Variable**	**Measure**	**Negative (0)**	**Positive (1)**	**P-value**

Region (n = 128)	1	6	23	0.061
		
	2	8	19	
		
	3	4	12	
		
	4	1	13	
		
	5	0	23	
		
	6	2	17	

Breeding Herd Size (n = 128)	1(9 to ≤ 13)	12	24	0.012
		
	2 (14 to ≤ 23)	4	26	
		
	3 (24 to ≤ 32.5)	3	27	
		
	4 (32.6 to ≤ 142)	2	30	

History of Leptospirosis (n = 128)	No (0)	19	105	0.065
	
	Yes (1)	2	2	

Weak Calves 2003 (n = 90)	No (0)	16	66	0.168
	
	Yes (1)	0	8	

Bull Y/N (n = 125)	No (0)	9	29	0.121
	
	Yes (1)	11	76	

Stock Bull (n = 125)	No (0)	11	30	0.021
	
	Yes (1)	9	75	

Hired/Borrowed Bull (n = 99)	No (0)	15	80	0.076
	
	Yes (1)	2	2	

Buy Rep Breed Animals (n = 118)	No (0)	15	63	0.093
	
	Yes (1)	3	37	

Grazing ≤ 830 acres (n = 124)	No (0)	19	74	0.024
	
	Yes (1)	1	30	

Grazing acres (n = 124)	1 (5 to ≤ 37)	7	25	0.144
		
	2 (38 to ≤ 60)	7	25	
		
	3 (61 to ≤ 86)	5	24	
		
	4 (87 to ≤ 830)	1	30	

%Grazing Wet (n = 121)	1 (0 to ≤ 3.1)	2	29	0.154
		
	2 (3.2 to ≤ 16.9)	4	26	
		
	3 (17 to ≤ 40)	6	25	
	
	4 (41 to ≤ 100)	8	21	

Straw-Bed Shed (n = 95)	No (0)	11	46	0.051
	
	Yes (1)	2	36	

Cows and Heifers Separate at Calving (n = 19)	No (0)	1	0	0.003
	
	Yes (1)	1	17	

Out-Winter Fed in Fields (n = 30)	No (0)	1	14	0.142
	
	Yes (1)	4	11	

Univariate statistical analysis, to a level of P < 0.05, was carried out on the 14 significant variables from the Chi-squared analysis. The results are outlined in Table 4.

**Table 4:** Significant Variables at P < 0.05, or Variables Approaching Significance only, following Univariate Analysis to assess the relationship between Herd Leptospirosis Status and 14 Variables in 128 Herds

Variable	Odds Ratio	95% Confidence Interval		P value
			
		Lower	Upper	
**Region (1,2,3,4,6) No 5** **excluded**	1.378	1.063	1.786	***0.016***
**Breeding Herd Size (Quartiles)**	2.039	1.252	3.322	***0.004***
**Stock Bull**	3.056	1.150	8.120	***0.025***
**Grazing Acres (Quartiles)**	1.607	1.013	2.548	***0.044***
**% Wet Land Grazed (Quartiles)**	0.588	0.368	0.940	***0.026***
**Straw-Bed Sheds**	4.304	0.897	20.658	**0.068**

Following univariate statistical analysis, there were 5 variables that showed a clear association with herd leptospirosis status (P < 0.05) - Region, Breeding Herd Size, Stock Bull, Grazing Acres and% Wet Land Grazed. The variable Straw-Bed Shed approached significance only.

The Correlation Coefficient (R) for Bull Y/N & Stock Bull was 1, implying internal confounding between these two variables. As both variables could not be included together in the multivariate analysis the variable Stock Bull was removed.

A backward selection model at P < 0.05 with all variables included was used for multivariable modelling. Only Region and Breeding Herd Size were retained in the model (Table 5).

**Table 5:** Results of multivariate analysis of the relationship between Region and Breeding Herd Size and prevalence of leptospiral infection in 128 Herds

Variable	Odds Ratio	95% Confidence Interval		
			
		Lower	Upper	P value
Region 1 (ref)	1			
Region 2	0.33	0.08	1.45	0.143
Region 3	0.72	0.13	3.98	0.709
Region 4	0.92	0.08	10.46	0.947
Region 6	1.16	0.18	7.58	0.873
Breeding herd size 9-≤ 13 (ref)	1			
Breeding herd size 14-≤ 23	5.47	1.16	25.92	***0.032***
Breeding herd size 24-≤ 32.5	3.88	0.84	17.90	0.083
Breeding herd size 32.6-≤ 142	7.08	1.17	42.77	***0.033***

Breeding herd size was found to be a statistically significant risk factor for leptospirosis in Irish suckler herds. The odds of a herd being positive for leptospiral infection were 5.47 times higher (P = 0.032) in herds with 14 to 23 breeding animals compared with herds with ≤ 13 breeding animals, adjusting for Region, and 7.08 times higher (P = 0.033) in herds with 32.6 to 142 breeding animals. In relation to the breeding herd size 24-32.5 category, breeding herd size only approached significance as a risk factor for herd seropositivity to *L*. Hardjo (P = 0.083). Despite the variable Region being retained in the model, there was no significant difference in the Regions relative to Region 1 (reference region).

## Discussion

This is the first risk factor survey of leptospiral infection due to Hardjo in unvaccinated suckler herds in the Republic of Ireland. Other risk factor studies have been published in the literature worldwide, but they refer mainly to dairy herds. The current study was carried out in conjunction with a seroprevalence survey of *Leptospira *Hardjo in unvaccinated Irish suckler herds [25]. This seroprevalence study found that there was widespread exposure to *Leptospira *Hardjo in Ireland, with a herd prevalence of 82.29% (n = 288) and an individual animal true seroprevalence, of 41.75% (n = 5366). There was a statistically significant association between increasing median breeding herd size by region and within-herd prevalence (P < 0.001) [25]. Region was also associated with seropositivity to Hardjo, with South-East Leinster and North Munster (Regions 5 and 3, respectively) having a particularly high herd and individual animal seroprevalence. This risk factor study was carried out to gather more in-depth details from the herds that were ELISA-tested, including details of animals, methods of housing, husbandry practices and drainage, so that other risk factors for herd seropositivity to *Leptospira *Hardjo in suckler herds could be ascertained. An additional objective was to investigate if the established risk factors for leptospirosis in dairy herds, including the presence of a river; sheep on the farm; the use of a bull; the purchase of cattle; and herd size [22], were relevant for Irish suckler herds.

### Key results

The key result to emerge from this risk factor study was the statistically significant relationship between breeding herd size and herd leptospirosis status. The risk of a herd being classified as "infected" (positive) due to *Leptospira *Hardjo was found to be statistically much higher in larger herds. The variables Region; use of a Stock Bull; Grazing Acres; and Percentage of Wet land Grazed on the farm; were statistically significant following univariate analysis only.

### Interpretation

The main finding of this study was the relationship between breeding herd size and herd prevalence to *Leptospira *Hardjo. This has been a common finding among most epidemiological studies into leptospirosis in beef and dairy herds [22,24,39,40]. It must be noted, however, that the 95% confidence intervals of the odds ratios relating to breeding herd size in this study are extremely wide (Table 5). This implies that while breeding herd size was found to be statistically significant by the multivariate model, the strength of this association between herd seropositivity to *Leptospira *Hardjo and breeding herd size may vary. A positive association between herd size and increased individual animal seroprevalence has also been reported previously for Hardjo infection in cattle [9,25,41]. The reason for this association is most likely a quantitative one, relating to the increased risk of exposure to disease in general in larger herds, resulting in the correlation between herd size and disease being common to a wide variety of diseases. Infections can be transmitted more easily and persist for longer in larger intensive herds [42,43]. This is supported by the finding that clinical disease due to leptospirosis was reported much more commonly in vaccinating herds in this study. It is unclear why herds in the 24-32.5 BHS category did not yield a significant association with seropositivity to *Leptospira *Hardjo, as there is a similar regional spread of "infected" herds between the different BHS categories as well as an approximately equal percentage of herds seropositive. However, even with due consideration given to the wide 95% confidence intervals around the odds ratios, there is clear statistical evidence that herd size is a significant risk factor for *Leptospira *Hardjo in Irish suckler herds.

Region appears to be a risk factor for bovine leptospirosis. Although differences in herd prevalence by Region were not significant on multivariate statistical analysis in this study, it was significantly associated on univariate analysis. In addition, Region was a significant risk factor for both herd and individual animal seroprevalence in the accompanying seroprevalence study into *Leptospira *Hardjo in Irish suckler herds [25] and it was the view of the authors that the high prevalence occurring in the South East of Ireland was related directly to the larger suckler herd sizes in this region. Regional variations in prevalence have been reported in other studies (both beef and dairy) also: in Switzerland [44], Australia [45], Mexico [46,47] and the USA [48,49]. Collectively, those authors reported a range of possible factors for the regional differences, including soil type, mean temperature and herd management practices. However, all of these studies involved a number of leptospiral serovars as well as Hardjo. As cattle are the maintenance host for *L*. Hardjo, environmental influences such as soil type, rainfall and mean temperature are unlikely to contribute to the regional variation in Hardjo prevalence in Ireland [2]. The fact that the variables Region; Grazing Acres;% Wet land Grazed and Stock Bull were all significant following univariate statistical analysis (P < 0.05) is interesting given the significance that was attributed to similar variables in other studies [4,9,20-24,39,40,44-47]. Also, a much higher percentage (57%) of vaccinating herds were operating an open herd policy and buying in animals. From a biosecurity and herd health perspective, it would be advisable to consider these variables as secondary risk factors for leptospirosis in suckler herds.

The limitations of study size and missing data may have contributed to variables being discarded by the multivariate model. Another possible reason for the failure to demonstrate truly significant associations between herd seropositivity to *Leptospira *Hardjo and well recognised risk factors from other studies (primarily dairy) [4,9,20-22] could be the different epidemiology of leptospirosis in beef and dairy herds [50]. In an Irish context, it appears that calves, reared alongside carrier cows, are exposed to Hardjo at a young age and are already seropositive prior to 12 months of age [25]. This is in contrast to findings in epidemiological studies in dairy herds where heifers are much more likely to be immunologically naïve on entering the milking herd [50]. From that point of view, the major risk factor in suckler herds would be the presence of a number of carrier animals in the herd (not assessed in this study), of which there are likely to be more in a larger herd, correlating well with the significance attributed to breeding herd size in this study.

It would appear that the risk factors for bovine leptospirosis vary widely in different parts of the world, and that this local epidemiological knowledge together with knowledge of the infecting serovars, is very important from a herd health and disease control point of view. For example, in the USA, a greater likelihood of infection with *L. borgpetersenii *serovar Hardjo was found in herds in California, Florida, Mississippi, Missouri, South Dakota and Texas, with higher mean annual temperatures and longer breeding seasons [49]. In Rio de Janeiro, the main risk factor associated with seropositivity to bovine leptospirosis was co-grazing with other species, mainly pigs. The absence of, or infrequent, veterinary assistance was also suggested to be associated with the overall seroprevalence to leptospirosis [51].

### Study limitations

One of the main limitations of studies of this kind is the presence of questionnaire response bias. The overall questionnaire response rate for the study was 49.06%. In mailed and self-completed questionnaires, response rates tend to be low--50% is not uncommon, and this value can be as low as 10% [52,53]. In many of the published studies involving a questionnaire aimed at determining risk factors for bovine leptospirosis, the questionnaire was carried out on the farm on the day of blood sampling [4,46,49], thereby negating the need for mailed questionnaires. An indicator of questionnaire response bias in this case is the fact that all the vaccinating herds (n = 21) responded to the questionnaire making up 13.38% of respondees. However, this is twice the percentage of vaccinating herds in the overall study (21/320) at 6.5%, indicating that prior knowledge or recognition of the disease contributed to the decision of many farmers to respond to the questionnaire.

A definite limitation of most studies is sample size--the higher the sample size, the more power that can be attributed to the findings of the study. In this risk factor study, the number of herds involved was 128. This compares favourably to the number of herds declared in some other published risk factor studies in the United Kingdom [22] (n = 78 beef and dairy herds); in Brazil [41] (n = 21 dairy herds) and [51] (n = 13 dairy herds), Spain [4] (n = 134 beef herds); the USA [49] (n = 72 beef herds) and in Tanzania [39] (n = 130 beef and dairy herds). A recent study which investigated the prevalence of antibodies to *Leptospira interrogans *serovar Hardjo in Irish dairy herds [24] involved 347 herds. The combined nature of the risk factor and seroprevalence studies in this project means that, while sample size may be a limitation to some degree, it should not have seriously affected the outcome and significance of the findings.

A third limiting factor was that of missing values. There was marked variability in the completion of questionnaires by each herdowner. Many of the questionnaires were fully completed, but many were also incomplete. To deal with the problem of missing values, it was decided to include incomplete records in the analysis and allow the statistical package to compensate accordingly. The advantage of this approach was the ability to maximize the dataset for statistical analysis, thereby strengthening the power of our findings.

### Implications for the Irish beef industry

The present study has significant implications for farmers and veterinary practitioners/herd health consultants when evaluating the impact of *Leptospira *Hardjo on Irish suckler farms. The risk of disease due to leptospirosis is likely to be much higher in larger suckler herds, particularly in the East of the country. Informed risk analysis is the key to successful decision making in relation to infectious disease control on farms. Noordhuizen (1996) stressed the importance of referring to the most recent literature in order to successfully implement herd health (HACCP-based) control as it applies to infectious disease at farm level [54]. The comparison of vaccinating and unvaccinated herds in this study suggests that, currently, the most common reason for vaccination against leptospirosis in Irish suckler herds is in response to a disease outbreak. Farmers, their veterinarians and other advisors should continue to consider leptospirosis among the main causes of infectious reproductive losses in Irish suckler herds, and take relevant steps to reduce exposure and minimise disease. It will be important to relate the findings of this study to Irish suckler farmers, through educational bodies and bodies working towards the improvement of animal health and welfare throughout the country, e.g. Animal Health Ireland (AHI).

Leptospirosis is a well-recognized zoonosis although disease due to *L*. Hardjo is usually subclinical, with 90% cases presenting as a flu-like illness [19]. In the time period between 1990 and 1996, the absolute incidence of leptospirosis due to *L*. Hardjo in the South Eastern Health Board (SEHB) was nearly 3.0/million which is double the national average and seven times the incidence in Great Britain [19]. This correlates also with the increased seroprevalence of *L*. Hardjo in the suckler and dairy cattle population in this region [24,25]. A survey from Northern Ireland in 1990 showed an 8.1% prevalence in farmers with *L*. Hardjo (48%) being the most common [18]. In a serological survey of 53 Irish agricultural workers in 1984, only *Leptospira *Hardjo was detected in just 3.8%. Of the thirteen serological cases in the SEHB (from a total of 28) whose occupation was determined, eight were in at-risk occupations: four were farmers, one a vet, one a butcher, one was a fencing contractor and another, a waterworks engineer [19]. The same zoonotic risk does not apply in a suckler herd compared to a dairy herd. Dairy farmers are at most risk from urine splashing in the parlour. However, suckler farmers and veterinary practitioners must continue to take correct precautions when calving suckler cows and when dealing with vaginal discharges.

## Abbreviations

AHI: Animal health Ireland; BHS: Breeding herd size; CMMS: Cattle movement monitoring system; CSO: Central statistics office; ELISA: Enzyme linked immunosorbent assay; HACCP: Hazard analysis critical control point; *L: Leptospira; *Lepto: Leptospirosis; LPS: Lipopolysaccharide; Mab: Monoclonal antibody; N: Number; NR: Herds that did not respond to the questionnaire; P: Probability value; R: Correlation coefficient; RF: Herds involved in the risk factor study; SEHB: South eastern health board; SP: Herds involved in the Seroprevalence study; USA: United States of America; *X*^2^: Chi-Squared test.

## Competing interests

None of the authors has any financial or personal relationships that could inappropriately influence or bias the content of the paper. This research was commissioned and funded by MSD, a pharmaceutical company that manufactures and sells a vaccine against *Leptospira *Hardjo. However, this has not influenced the nature of the study, the results of the study or the conclusions of the study.

## Authors' contributions

EGR collected the serum samples, performed the ELISA tests and was the primary author of the paper. NL provided specific expertise in the field of bovine leptospirosis, as well as acting as one of the supervisors of the project. MLD and LOG provided expertise in relation to the statistical interpretation of data and the structured writing of the paper, in addition to supervising the project. SM acted as principal supervisor and provided expertise in the area of epidemiological research. All authors read and approved the final manuscript.
